# *Isoetes mississippiensis*: A new quillwort from Mississippi, USA

**DOI:** 10.3897/phytokeys.74.10380

**Published:** 2016-11-08

**Authors:** Peter W. Schafran, Steven W. Leonard, Rebecca D. Bray, W. Carl Taylor, Lytton J. Musselman

**Affiliations:** 1 Department of Biological Sciences, Old Dominion University, Norfolk, Virginia 23529-0266; 2 Wiggins, Mississippi 39577; 3 Department of Botany, National Museum of Natural History, Washington, DC 20560-0166

**Keywords:** Isoetes, Isoetaceae, lycophyte, quillwort, Mississippi

## Abstract

*Isoetes mississippiensis* S.W. Leonard, W.C. Taylor, L.J. Musselman and R.D. Bray (Isoetaceae, Lycopodiophyta) is a new species known from two sites along tributaries of the Pearl River in southern Mississippi. This species is distinguished from other species in the southeastern United States by a combination of character states including a basic diploid (2n=22) chromosome count, laevigate megaspores, and a narrow velum covering less than one-third of the adaxial sporangium wall.

## Introduction

*Isoetes* (Isoetaceae) is a cosmopolitan genus of heterosporous lycophytes containing 200–300 species (Hickey et al. 2003; Troia et al. 2016). Lycophytes have an extensive fossil record dating from the Devonian and a morphology so conserved that members of the genus *Isoetes* are recognized in the Triassic (Pigg 2001). Extant species are widely distributed from the tropics to the sub-arctic (Troia et al. 2016). They range in habitat from evergreen aquatics to seasonal terrestrials. Resembling a tuft of chives or grass, they are easily overlooked in the field.

In spite of their antiquity, widespread distribution, and diverse ecological adaptations, *Isoetes* species are remarkably uniform in their morphology. Plants appear simple in form with a lobed subterranean rootstock producing a tuft of linear sporophylls above and below roots along a groove between the lobes. This apparent morphological simplicity makes it easy to recognize a member of the genus, but difficult to distinguish species. Earlier taxonomists relied primarily on habitat, megaspore texture, and megaspore size to separate taxa (Engelmann 1882; Pfeiffer 1922; Reed 1965; Boom 1982). More recently, chromosome counts and molecular markers have been used to further define taxa and infer their phylogeny (Taylor et al. 1993, Hoot et al. 2004; Heafner and Bray 2005; Rosenthal et al. 2014).

Ornamentation and size of megaspores and microspores are important morphological features used to identify species of *Isoetes*. Pfeiffer (1922) erected four sections based on the megaspore ornamentation types cristate, echinate, reticulate, and tuberculate. While these sections are no longer recognized as having phylogenetic value in the genus, the emphasis on macro-ornamentation for identification remains (Brunton 2015). Several categories for megaspores (cristate, echinate, laevigate, psilate, reticulate, rugulate, and tuberculate) and microspores (aculeate, cristate, echinate, laevigate, and psilate) are accepted, though there can be gradation between categories (Taylor et al. 1993; Musselman 2002). Micro-ornamentation of megaspores and microspores is sometimes recognized, but has not been included in any recent taxonomic treatments of the genus (Reed 1965; Boom 1982; Taylor et al. 1993; Brunton 2015). Generally, megaspore size increases with ploidy level (Pereira et al. 2015; Brunton 2015).

The habitat of species of *Isoetes* can be fairly specific and is often used in taxonomic treatments (Engelmann 1882; Reed 1965; Taylor et al. 1993; Brunton 2015). Species are generally segregated as aquatic, amphibious, or terrestrial, based on the proportion of their growing season spent in water (Engelmann 1882; Taylor et al. 1993). Some species occur only in certain habitats, such as rock pools, calcareous glades, oligotrophic lakes, and swamp forests. Widespread species such as *Isoetes melanopoda* Gay and Durieu (*s.s.*) and *Isoetes engelmannii* Braun have more varied habitat preference (Taylor et al. 1993; Brunton 2015).

Characteristics of sporophylls and rootstocks of *Isoetes* may also provide taxonomic information, though the utility of some of these features is questionable. Velum coverage of the sporangium, sporangium shape, sporangium wall coloration, and sporophyll length, number, color, and shape are sometimes used for species identification, but these character states can be subtle and it is unclear how they may be influenced by environmental conditions (Engelmann 1882, Pfeiffer 1922; Reed 1965, Boom 1982, Taylor et al. 1993, Brunton 2015). Cultivated plants often appear different than those *in situ*, and spore development, photosynthetic pathways, and gene expression are significantly altered by water conditions (Brunton 2015; Yang and Liu 2015; Yang and Liu 2016). However, the *gestalt* formed from the combination of these characters usually leads experts to accurate field identification.

While searching for populations of *Isoetes louisianensis* in southwestern Pearl River Co., MS, in the spring of 1996, one of us (Leonard) discovered a population of *Isoetes* that did not appear to be *Isoetes louisianensis* or any other known species. These plants had very long and numerous sporophylls bearing megaspores with a smooth surface rather than an irregularly reticulate texture that is typical of *Isoetes louisianensis* megaspores. In addition, the megaspores of this plant were noticeably smaller than those of *Isoetes louisianensis*. Further investigation turned up a second population downstream in Lotts Creek. Both of these waterways are tributaries of the Pearl River, converging near Picayune, MS.

## Methods

Field work was performed in 1996, 1998, and 2013 to obtain specimens for further study. Specimens were deposited in the Old Dominion University herbarium (ODU). Length and width of the rootstock, sporophylls, and sporangia were measured for 10 individuals. Megaspores and microspores were removed from sporangia, cleaned by sonication in distilled water for 90 seconds, and dried on a slide warmer at maximum temperature (approximately 60°C). Light images were captured using a Nikon SMZ800 stereomicroscope with attached Digital Sight camera, and measurements made within the Digital Sight control panel. Spores were prepared for scanning electron microscopy by coating with 25 nm of gold-palladium using a Cressington high resolution sputter coater (Cressington Scientific Instruments Ltd.). Imaging was performed on a Zeiss EVO MA 15 scanning electron microscope. Chromosome counts were determined by root tip squashing as described in Heafner and Bray (2005). Site descriptions were prepared and lists of associated species were made.

## Results

Analysis of morphological characters, chromosome counts, and ecological evaluation leads us to conclude our collections represent an undescribed species of *Isoetes*.

### Isoetes mississippiensis

Taxon classificationPlantaeIsoetalesIsoetaceae

S.W. Leonard, W.C. Taylor, L.J. Musselman & R.D. Bray sp. nov.

urn:lsid:ipni.org:names:77158528-1

#### Type.

USA. Mississippi: Lotts Creek (30.57396°N, 89.76196°W, elevation 14 m), 18 June 2013, *P. Schafran MS-08 L. Musselman, S. Leonard, W. Taylor, M. Alford, and D. McNair* (holotype: US; isotypes: MO, NY, ODU, USMS).

#### Description.

Plants amphibious in and along persistent streams. Rootstock subglobose, bilobed, brown, 0.5−1.0 cm long, 1.0−1.5 cm wide. Roots dichotomously branched. Sporophylls (leaves) linear, bright green, darkening with age, pale toward base, spirally arranged, erect to spreading, up to 40 cm long and 2.0 mm wide at mid-length, in tufts of ca. 20, semi−terete with adaxial surface flattened, becoming more terete distally, with translucent alae ca. 1 mm wide extending along lateral edges from base to ca. one-quarter leaf length, tapering gradually toward apex, abruptly dilated and spatulate toward base where streaks of brown pigmented cells are often evident on pale outer surface of leaf base. Ligule triangular, ca. 1 mm long. Sporangium ovate, most 4−10 mm long, most 4−5 mm wide, adaxial wall spotted to streaked with scattered clusters of brown pigmented cells. Velum incomplete, covering less than one third of sporangium wall. Megaspores globose, white, trilete, macro-ornamentation laevigate with echinate micro-ornamentation, ca. 280−380 µm in diameter, averaging ca. 340 µm. Microspores broadly fusiform, macro-ornamentation echinate with bacillate micro-ornamentation, pale brown in mass, monolete, 25−30 µm long.

#### Morphology.

**Figure 1.**
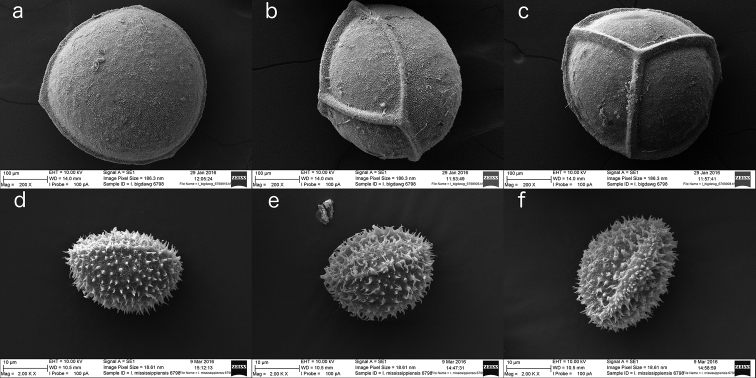
SEMs of megaspores (**a, b, c**) and microspores (**d, e, f**) of *Isoetes mississippiensis* displaying distal (**a, d**), equatorial (**b, e**), and proximal (**c, f**) views. Megaspores from Schafran MS-08, microspores from Taylor 6798. Megaspore magnification 200×; microspore magnification 2000×.

**Figure 2.**
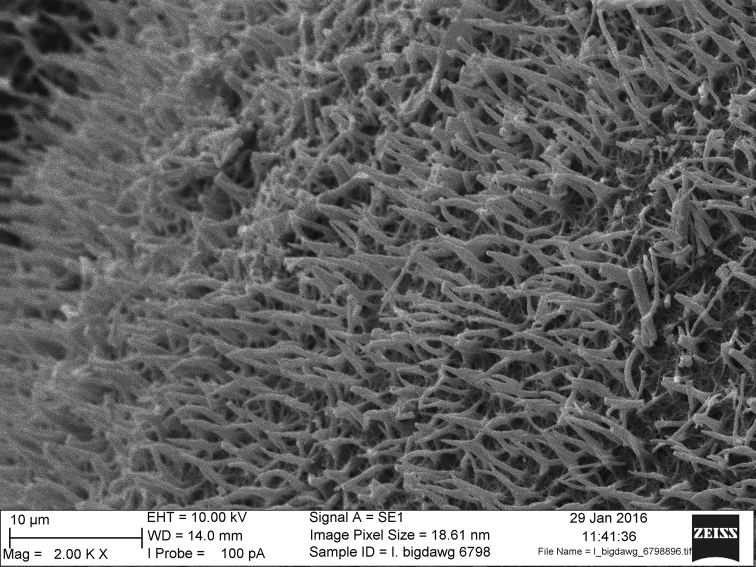
SEM detail of megaspore micro-ornamentation. Magnification 2000×.

**Figure 3.**
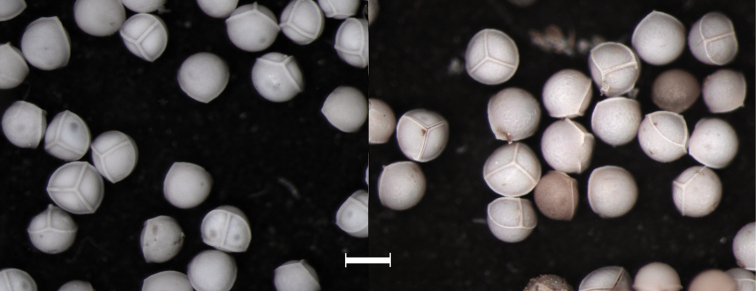
Light microscope image of megaspores of *Isoetes mississippiensis* from Schafran MS-07 (left) and MS-08 (right). Magnification 63×. Scale bar = 0.3 mm.

Rootstocks of all specimens examined vary in length from 0.5−1.0 cm and in width from 1.0−1.5 cm. All rootstocks are subglobose in shape and bilobed. Sporophylls reach a maximum length of 40 cm and maximum width of 2.0 mm at mid-length. Sporangia are 4−10 mm long and 4−5 mm wide. Megaspores are laevigate with echinate micro-ornamentation (Figures 1, 2, 3). Diameter of megaspores varies from 280−380 µm, with an average of 340 µm. Microspores are echinate with bacillate micro-ornamentation, and are 25−30 µm long (Figure 1).

#### Cytology.

Chromosome counts show individuals of *Isoetes mississippiensis* to be diploid (2n=22).

#### Ecology.

**Figure 4.**
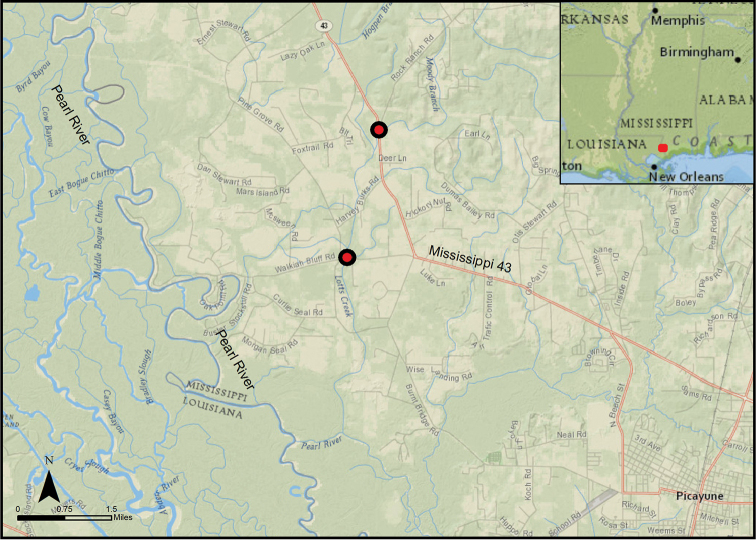
Map showing two localities of *Isoetes mississippiensis*. Inset: Map of Mississippi with detail area highlighted. Map created using ArcGIS software (Esri).

*Isoetes mississippiensis* occurs in sluggish, persistent streams in southern Mississippi (Figure 4). At the Moody Branch locality, the maintained right-of-way of Mississippi Highway 43 allows abundant sunshine to reach the stream and adjacent wetlands. Small bushes and saplings of titi (*Cyrilla racemiflora*) and red maple (*Acer rubrum*) are periodically cut down and allowed to fall in the stream. Sediment and detritus provide anchors for herbaceous growth of sedges, rushes, and coarse grasses (*Rhynchospora inexpansa*, *Juncus* spp., *Erianthus giganteus*, *Panicum* spp.). In the shallow water stream margin is *Iris virginica*. The woodland edge is suitable habitat for crossvine (*Bignonia capreolata*) and rattan vine (*Berchemia scandens*). Upstream where a defined channel is present the overstory consists of swamp black gum (*Nyssa biflora*), laurel oak (*Quercus laurifolia*), red maple, and encroaching loblolly pines (*Pinus taeda*). Shrubs in the understory are Elliott’s blueberry (*Vaccinium elliottii*), yaupon (*Ilex vomitoria*), and titi. In the upper reaches of Moody Branch, the channel is braided and the water sluggish, more typical of a swamp black gum forest with Rankin’s jessamine (*Gelsemium rankinii*), Virginia willow (*Itea virginica*), and dog hobble (*Viburnum nudum*).

After flowing west for several kilometers, Moody Branch turns sharply south just west of Mississippi Highway 43 and eventually merges with Lotts Creek. The forested wetland adds pond cypress (*Taxodium ascendens*) and a dense shrub understory with *Smilax laurifolia*. At the Walkiah Bluff Road crossing of Lotts Creek disturbance has been severe, yet *Isoetes mississippiensis* has revegetated new habitat in the roadside ditch north of the road and on sandbars.

#### Etymology.

This species is named for the state of Mississippi, its only known locality.

#### Specimens examined.

Leonard 9393, 9 March 1996 (MMNS); Leonard 9395, 22 March 1996 (MMNS); Leonard 9831, 2 June 1997 (MMNS); Leonard 12405, 12 May 2011(ODU); Leonard 12406, 12 May 2011 (ODU); Musselman with Taylor, 98908, 17 October 1998 (ODU); Bolin JB-MS-01, 9 January 2009 (ODU); Schafran MS-07, 18 June 2013 with Musselman, Leonard, Taylor, and Alford (MO; NY; ODU; USMS); Schafran MS-08, 18 June 2013 with Musselman, Leonard, Taylor, and Alford (US; ODU); Taylor 6798, 18 June 2013 with Musselman, Leonard, Schafran, and Alford(US).

## Discussion

Evaluation of the morphological and cytological features of *Isoetes mississippiensis* shows it to be distinct from all other taxa in the southeastern US. In the coastal plain of the Gulf Coast states, nine other species are known: *Isoetes appalachiana*, *Isoetes boomii*, *Isoetes flaccida* *s.l.*, *Isoetes hyemalis*, *Isoetes louisianensis*, *Isoetes melanopoda* *s.l.*, *Isoetes microvela*, *Isoetes texana*, and *Isoetes valida* (Singhurst et al. 2011; Weakley 2015; Brunton 2015). A basic diploid chromosome count (2n=22) plus laevigate megaspore ornamentation separates *Isoetes mississippiensis* from all these taxa except *Isoetes texana* and occasionally *Isoetes melanopoda*. These species may be further separated by presence/absence of phyllopodia, difference in megaspore size, and velum coverage (Table 1). Additionally, the habitats of these species are quite different. *Isoetes mississippiensis* occurs along persistent streams, while *Isoetes texana* is found in freshwater ponds and interdunal swales and *Isoetes melanopoda* grows in wet prairies, soil pockets on rock outcrops, and woodland depressions (Table 1; Singhurst et al. 2011; Taylor et al. 1993).

**Table 1.** Comparison of Gulf Coastal Plain *Isoetes*.

Character	*Isoetes mississippiensis*	*Isoetes texana*	*Isoetes flaccida* *s.l.*	*Isoetes melanopoda* *s.l.*	*Isoetes valida*
Ploidy	2n=22	2n=22	2n=22	2n=22	2n=22
Habitat	Persistent streams	Persistent freshwater ponds, interdunal swales	Springs, stream bottoms, river bottoms, ditches	Ephemeral wet prairies, open graminoid swales, woodland pools, soil pockets on rock outcrops	Woodland seepages
Megaspore Ornamentation	Laevigate	Smooth to obscurely rugulose	Low tubercules to broad, interconnected mounds	Low tubercles or ridges	Broken reticulate
Megaspore Size (mm)	280–380 (x=340)	350–405 (no mean reported)	250–500 (no mean reported)	280–440 (x=380–410)	x=450
Microspore Ornamentation	Spinulose/echinate	Papillose	Papillose	Spinulose/echinate	Spinulose/echinate
Microspore Size (mm)	25–30	25–30	25–33	20–30	27
Velum Coverage (%)	15–33	100	80–100	5–15	45–70
Character	*Isoetes louisianensis*	*Isoetes hyemalis*	*Isoetes appalachiana*	*Isoetes boomii*	*Isoetes microvela*
Ploidy	2n=44	2n=44	2n=44	2n=66	2n=66
Habitat	Creeks, streams	Blackwater streams	Creek banks, woodland pools, lakes	Slow-flowing woodland streams	Persistent streams in deciduous swamp forests
Megaspore Ornamentation	Irregularly reticulate	Broken reticulate to sub-echinate	Broken reticulate	Cristate to reticulate	Densely reticulate with irregular crests and thin tubercles
Megaspore Size (mm)	500–625 (no mean reported)	400–580 (=522)	450–611(=534)	460–610 (no mean reported)	=527
Microspore Ornamentation	Spinulose/echinate	Spinulose/echinate	Psilate to low tuberculate	Papillose/aculeate	Psilate to low tuberculate
Microspore Size (mm)	25–35	20–31	29–32	25–30	30
Velum Coverage (%)	<50	10–20	20–25	30–50	10

### Key to the Diploid Species of *Isoetes* of the Gulf Coastal Plain of the United States

**Table:** 

1	Megaspores psilate to laevigate, rarely low tuberculate or low rugulate	**2**
2	Plants at least sometimes with darkened, often sclerified, brown-black leaf bases; velum coverage generally <15%	***Isoetes melanopodas.l.***
2'	Plants never with darkened leaf bases; velum coverage usually >15%	**3**
3	Megaspores 280-380 mm; velum coverage 15-30%	***Isoetes mississippiensis***
3'	Megaspores 350-405 mm; velum coverage 100%	***Isoetes texana***
1'	Megaspores tuberculate, reticulate, cristate, or rugulate	**4**
4	Velum coverage 75-100%; microspores papillose	***Isoetes flaccidas.l.***
4'	Velum coverage less than 75%; microspores echinate	**5**
5	Megaspore ornamentation of tubercles or ridges; velum coverage less than ca. 25%	***Isoetes melanopodas.l.***
5'	Megaspore ornamentation broken reticulate; velum coverage between ca. 25 and 75%	***Isoetes valida***

## Conservation

*Isoetes mississippiensis* is known from only two locations along approximately 2 miles of the Lotts Creek—Moody Branch waterway. Neither of these populations is located on preserved land. Extensive field work is needed to search for additional populations in the nearby Pearl River Wildlife Management Area and Bogue Chitto National Wildlife Refuge.

## Supplementary Material

XML Treatment for Isoetes mississippiensis
